# Context‐dependent female preference for multiple ornaments in the bearded reedling

**DOI:** 10.1002/ece3.1903

**Published:** 2016-01-08

**Authors:** Matteo Griggio, Herbert Hoi, Barbara Lukasch, Andrea Pilastro

**Affiliations:** ^1^ Dipartimento di BiologiaUniversità di PadovaVia U. Bassi 58/BI‐35131PadovaItaly; ^2^ Department of Integrative Biology and EvolutionKonrad Lorenz Institute of EthologyUniversity of Veterinary Medicine of ViennaSavoyenstrasse 1/AA‐1160ViennaAustria

**Keywords:** Multiple traits, ornaments, preference dilution effect, sexual selection

## Abstract

While it is well established that females prefer to mate with well‐ornamented males, the influence of perceptive and cognitive processes on the expression of female mate choice is still poorly known. It has been suggested that the female perception of a male's attractiveness is not absolute, but depends on the other males with which he is compared that have been previously encountered (comparative evaluation). We investigated whether mate preference in bearded reedlings (*Panurus biarmicus*) is dependent on or independent of social context in relation to two different traits: beard and tail lengths. Each female had a choice between two to three males with different modifications of beard and tail. For each female, three different experiments were conducted (one binary and two trinary tests). We found that when females are presented with options that vary antagonistically with respect to two ornaments (binary test), some individuals prefer one trait while others the other trait. This indicates that in our bearded reedlings population exists a mate preference polymorphisms. Moreover, we found that the presence of a third stimulus, irrespective of the initial preference, reduced the strength of the initial preference – what we can call a “preference dilution effect.” Our results suggest that the female's choice may be constrained by her cognitive abilities when she is simultaneously presented with several options varying for two uncorrelated traits.

## Introduction

In animal kingdom, females are usually the more choosy sex, because of their higher initial investment due to anisogamy and the choice is frequently based on multiple rather than a single male trait (Andersson 1994; Iwasa and Pomiankowski 1994; Andersson and Simmons 2006). We still know little about the underlying decision making processes females use to select a mate. For example, we know little about which and how many different traits females may integrate and how they are weighed (Bateson and Healy 2005; Royle et al. 2008; Castellano 2009; Reaney 2009). During mate choice, females may be further faced to a variable number of potential mating partners. Depending on the number of quality features considered and the number of mating partners available, trait attributes to be considered may become rapidly difficult to process. Nonetheless one may predict that female decision making is optimized, which means that it follows rational and reproducible rules. Consequently, individuals are expected to (1) consistently choose the potential partner with the highest value and (2) the perception of the value of an individual is expected to be independent of other options available (e.g., Pyke et al. 1977). Indeed, models based on rational decision making predict that, given the relative preferences between two options, the addition of a third option should not influence the relative preference for the two main options (Luce 1959; Tversky and Simonson 1993). In contrast, several choice studies on humans (e.g., Huber et al. 1982; see Sedikides et al. 1999 for partner selection) and other animal species (e.g., Shafir 1994; Waite 2001; Shafir et al. 2002; Bateson et al. 2003; Scarpi 2011) indicate that perception of the magnitude of a stimulus is affected by comparison with other stimuli.

Females frequently encounter a number of potential mates, either sequentially or simultaneously, allowing comparisons among males. Preferences may be not be absolute but may depend on the attractiveness of other males a female has previously encountered or in the case of a colonial or lekking species, is simultaneously faced too. Thus, discarding the concept that females follow strict absolute trait values when choosing a mate may produce new more realistic insights into mate choice. There is in fact already evidence that females, when comparing males do not necessarily assign them according to absolute values, they are rather compared along different dimensional scales (Bateson and Healy 2005; Bailey 2011). This process is additionally complicated by the fact that individuals usually choose mates based on the assessment of multiple ornaments (Møller and Pomiankowski 1993; Flanagan et al. 2014; Freeman‐Gallant et al. 2014) which, at least in some cases, seems to provide information about different underlying qualities (e.g., Candolin 2003; Rivera‐Gutierrez et al. 2010; Hoi and Griggio 2011). Comparing several alternative mates differing by two or more uncorrelated traits is likely to be a difficult cognitive task and it has been proposed that the possible effect of cognitive constraints and perceptive distortions on female choice should not be ignored (Bateson and Healy 2005). Surprisingly, few studies have investigated the effects of modifying different ornaments and composition of the male choice set (Royle et al. 2008; Reaney 2009; Locatello et al. 2015). A recent study on great bowerbirds (*Ptilonorhynchus nuchalys*) suggests that cognitive constraints and perspective distortions may have strong implications for the evolution of particular traits under sexual selection (Kelley and Endler 2012a,b). In particular, the “asymmetrically dominated decoy” effect predicts that when a decision between two alternatives is based on two (or more) choice criteria (options), the presence of an asymmetrically dominated “decoy” increases the attractiveness of the “dominant” option. So, following this hypothesis, if a female has to select between two males (A and B) and the choice is based on two traits, for example, traits 1 and 2, the introduction of a third male (C) that is an asymmetrically dominated decoy (this male has a lower value than males A and B for the trait 1, but it is higher than male B for the trait 2) means the female should increase the preference for the dominant option – male A, in our case (Sedikides et al. 1999 and see fig 1 in Bateson and Healy 2005). This could result from the perceptual effects of altering the range of stimuli. In our case, male C has a lower value for trait 1 and therefore has the perceptual effect of reducing the subjective difference between A and B in trait 1. This could increase the overall value of A relative to B because male A has a higher value of trait 2 that becomes more relevant thanks to the presence of male C.

In line with this the question we focus in this study is whether evaluation of multiple traits changes with the possibilities – the number of potential mating partners. Our model system, the bearded reedling (*Panurus biarmicus*), seems to be appropriate to investigate the comparative evaluation of multiple male traits in relation to changing possibilities. Female bearded reedlings select their mates according to morphological characters such as beard length (Hoi and Griggio 2008) and tail length (Romero‐Pujante et al. 2002). By settling in colonies, females are simultaneously faced to a number of potential mates that differ in more than one ornament (Hoi and Griggio 2012). We investigated whether mate preference in bearded reedlings is dependent on or independent of the social context in relation to two different sexually selected traits: beard and tail lengths. Experimentally each female bearded reedling was given the choice between two or three males with different modifications of beard and tail. Thus, to keep one trait the same while altering the other, we manipulated the tail and the beard lengths to obtain four groups of males: (1) males with long tails and short beards; (2) males with long tails, as the previous group, but beards shorter than the previous group, (3) males with short tails and long beards, and (4) males with short tails, as the previous group, but beards shorter than the previous group. For each female, three different experiments were conducted (one binary and two trinary tests). If females are using an absolute choice, the addition of a third stimulus should not affect the female preference. On the contrary, if the addition of a third stimulus, a decoy, changes the relative preference for the most preferred stimulus, it would indicate a context‐dependent preference supporting the asymmetrically dominated decoy effect.

## Materials and Methods

### Mate choice protocol

We used 84 bearded reedlings (44 females and 40 males) captured from July to October at Lake Neusiedl (47°56′N, 16°45′E) and housed in captivity at the Konrad Lorenz Institute of Ethology (KLIVV, Vienna, Austria, 48°13′N, 16°17′E). They were captured with mist nets and transported to the KLIVV in cotton bags. To avoid possible previous experience of each other, males and females were collected at different locations and different times. All males were kept in the same housing room in singular cages (100 × 50 × 50 cm). The housing room was maintained at a constant temperature of about 20°C and on a 14/10‐h light/dark photoperiod. Females were kept in six outdoor aviaries (3.5 m × 3.5 m × 2.5 m; 7–8 females per aviary). Sexes were visually and vocally isolated until the start of the experiment and birds used in the experiment had no prior contact with each other (for more details, see Griggio and Hoi 2011). All aviaries were equipped with reed bushes, water, and a central feeder. Commercial food for insectivorous passerines and mealworms was provided ad libitum.

Morphological measurements were taken prior to the start of the experiments. Measurements were body mass (to the nearest 0.1 g), bill length (exposed culmen) maximum wing length, tarsus, and tail length (±0.01 mm; Svensson 1992). Beard length was calculated as the average of both beard stripes measured from the posterior bill end to the beard tip (±0.01 mm) with digital callipers (Hoi and Hoi‐Leitner 1997). Among the randomly selected males, tail and beard were modified irrespective of the initial length or other biometrical characteristics. To manipulate the two traits, we used the same methodology developed by Romero‐Pujante et al. (2002) and Hoi and Griggio (2008, 2011). Briefly, tail feather tips (except the outer tail feathers) and terminal beard feathers of all males were cut with scissors to a length of 50 mm and 9 mm, respectively. So, the two traits were adjusted to the same common magnitude for all males. Afterward, new tail feathers were glued and new beards painted onto each male according to the group they were in. For tail manipulation, four feathers were added to the original tail feathers (except the outer tail feathers), using small amounts of a strong instant glue. The overlapping glued surface was 5 mm^2^ (for more details, see Romero‐Pujante et al. 2002). Simultaneously, the two beards of each male were manipulated by painting the black and other (gray) feathers with black nail polish to the length desiderate (for more details, see Hoi and Griggio 2008, 2011). Observations after manipulation did not seem to stress the birds and they did not show any difficulties in flying or landing. All the manipulations were performed in the natural range of tail and beard lengths (Romero‐Pujante et al. 2002; Hoi and Griggio 2011).

In accordance with the objectives of our study, four groups of males were formed:

1. Males with long tails and short beards (long tail male: T‐male, hereafter): Tail: 95–100 mm with an average of 99 mm, beard: 12.2–13 mm with an average of 12.9 mm;
2. Males with long tails, as the T‐males, but beards shorter than all other groups. So, these males have a lower value than T‐males for the beard length, but the same value for the tail length (“decoy” of the T‐male: DT‐male, hereafter): Tail: 95–100 mm with an average of 99 mm, beard: 9.2–10 mm with an average of 9.9 mm;
3. Males with short tails and long beards (long beard male: B‐male, hereafter): Tail: 64–70 mm with an average of 67.8 mm, beard: 24.2–27 mm with an average of 26.6 mm;
4. Males with short tails, as B‐males, but beards longer than DT‐males and DB‐males, but shorter than B‐males. So, these males have a lower value than B‐males for the beard length, but the same value for the tail length (“decoy” of the B‐male: DB‐male, hereafter): Tail: 64–70 mm with an average of 67.8 mm, beard: 24–26.2 mm with an average of 24.4 mm).

**Figure 1:**
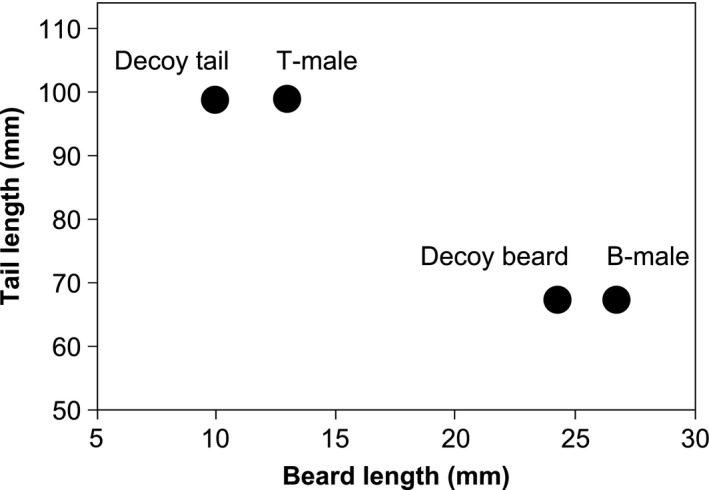
Diagram showing the position of the four groups of males used in the experiments relative to the two traits used (tail and beard length). Decoy tail male (DT‐male) is more attractive than long beard male (B‐male) for tail length but less attractive for beard length. Decoy beard male (DB‐male) is more attractive than long tail male (T‐male) for beard length but less attractive for tail length.

In this way, a DT‐male is more attractive than a B‐male for tail length but less attractive for beard length. A DB‐male is more attractive than a T‐male for beard length but less attractive for tail length (Fig. 1).

We conducted a female mate preference test using a four‐choice apparatus (2 m × 2 m × 0.5 m) which was positioned in a room similar to the housing room. The apparatus consisted of four‐choice chambers, separated by opaque dividers, at the four sides of the central choice chamber. An opaque divider was also set up on each of the four sides of the central chamber to avoid visual interaction between the four stimulus males (for more details, see Griggio et al. 2009, 2011). The dividers also prevented females from simultaneously observing two or more males. In one corner of the four dividers, an opening (14 × 14 cm) covered by a metal web allowed the female to observe the males in the side chambers (Griggio et al. 2009, 2011). During the experiment, the females could see the males through these holes but they could not physically interact. A perch was positioned in front of each of the four chambers. Perches had a line marked on them which corresponded to the limit from which a female could observe the male in the nearby compartment (for more details, see Griggio et al. 2009, 2011).

In accordance with the objectives of the study, females had a choice between two to three males with different modifications of beard and tail. For each female, three different experiments were conducted (one binary and two trinary tests). (1) Two choice experiment (binary test): females choose between two males with either long tail (T‐male) or long beard (B‐male); (2) decoy tail experiment (trinary test): females choose between three males – a T‐male, a B‐male, and a DT‐male; (3) decoy beard experiment (trinary test): females choose between three males – a T‐male, a B‐male, and a DB‐male. The three experiments were performed in variable order between March and April, and always during a different time of the day, from 9:00 am to 4:00 pm. Between the three experiments, there was a break of 1 week (7 days) for each female. Each female saw the same stimulus set of males, but the set of individual males was different for each female.

The chambers were randomly assigned to the stimulus males and one or two chambers were empty during the experiment, depending on the type of experiment: with two or three stimuli, respectively. At the beginning of a trial, choosing females and stimulus males were placed in their experimental chambers and allowed at least 30 min to acclimatize before the trial began. After that period, the opaque separators were removed and the position of the female was recorded every 1 sec for 30 min (Hoi and Griggio 2011, 2012; Lahaye et al. 2013). All trials were video‐recorded and then analyzed. For an estimator of proximity preference, we measured the time spent by a female on the part of the perch in front of a male's compartment (choice time). Preference was expressed as the proportion of time in front of each male over the total time in the choice area (e.g., Brooks 2000; Griggio et al. 2009, 2011). Once a trial ended, the stimulus males were returned to the housing cages and a standardized housing setting for all stimulus males was maintained throughout the experiment. Water and food were provided ad libitum during the experiments. After the experiment, all the birds were released in several outdoor aviaries (3.5 m × 3.5 m × 2.5 m), where most of them bred.

### Repeatability of mate preference in bearded reedlings

A basic assumption for our experiment on mate preference is some level of consistency in female preference. For this reason, the year before this set of experiments commenced, an experiment was performed with a different set of males and females to investigate the consistency of individual bearded reedling females' choices (Forstmeier and Birkhead 2004). Briefly, the same female (*N* = 15) was tested two times (2‐week intervals) with the same set of males (*N* = 30; two stimulus males with beard length manipulated) using an experimental setup similar to that described above. We determined the repeatability of each female's preference for male trait (long or short beard) between two trials following Lessells and Boag (1987), by calculating separate analyses of variance for each female with association time as the dependent variable and trial number as an independent variable. We found a high consistency of female preference (repeatability of time allocation by a female tested twice with the same set of males: *R* = 0.68, measurement error = 0.32, SE = 0.14).

### Statistical analyses

Statistical analyses were performed with SPSS 15.0 (Norušis 1993). All the results are presented as mean ± SE. All probabilities are two‐tailed. Data were checked to ensure that they met the requirements of parametric statistics. The prediction tested here was that the relative preference for the B‐males over the T‐males was influenced by the presence of a third stimulus (DT‐male or DB‐male). We therefore calculated the time females spent in front of each male and then used the relative preference for the tail male over the total time spent in front of the two main stimuli. We used a general linear mixed effects model with the relative time the females spent in front of the B‐male as the dependent variable. The random factor was female identity and the fixed factor was the set of stimuli (three levels: binary choice, DB‐male trinary, and DT‐male trinary). As we were interested in testing the effect of the decoy type relative to the binary female preference, we also calculated the difference between the relative preference for the tail male in the presence of each decoy and her preference for the tail male in the binary test.

### Ethics statements

Immediately after the experiment, all birds started breeding successfully suggesting that the housing conditions were appropriate and that the experimental birds remained healthy. Licenses to take and keep birds from the field were given by the Burgenländische Landesregierung (No. IV‐1253/38; IV‐1058/39; and 5‐N‐A1007/178, 5‐N‐A‐1007/367‐2009 based on the “Burgenländisches Naturschutzgesetz”: LGBI.Nr. 22/1980). The experiments reported in this article comply with current laws on animal experimentation in Austria and the European Union. This study was approved by the institutional ethics committee and the national authority according to § 8ff of Law for Animal Experiments Tierversuchsgesetz – TVG.

## Results

**Figure 2:**
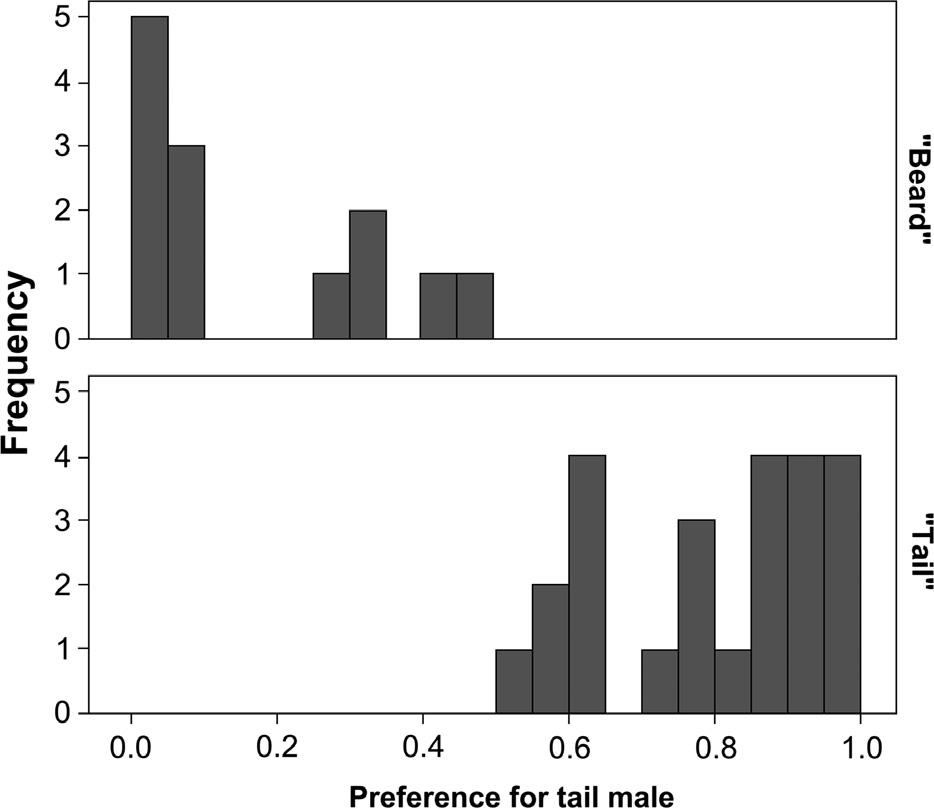
Distribution of the preference for the T‐male in the binary choice experiment. Females showing a preference for tail length (“Tail,” values above 0.5, *N* = 24) and for beard length (“Beard,” values below 0.5, *N* = 13) are shown in the upper and lower panels, respectively.

**Figure 3:**
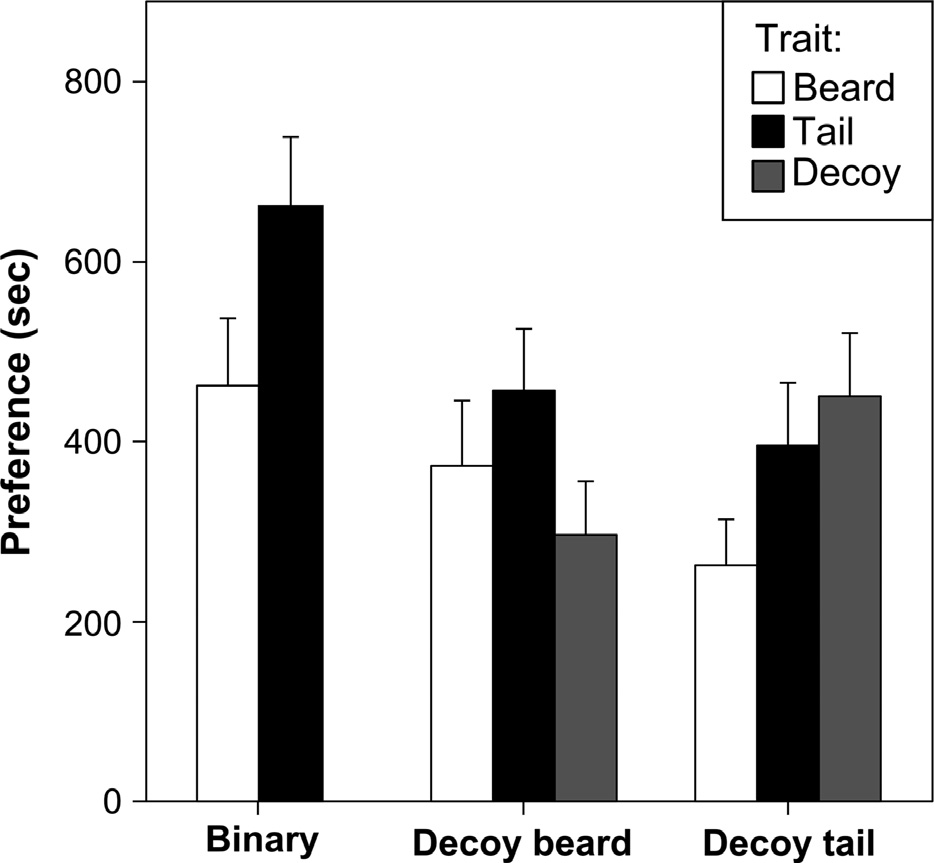
Absolute preference in seconds for the four kinds of males (T‐male, B‐male, DT‐male, and DB‐male) in the three different experimental trials (binary choice; trinary with the decoy for the beard; trinary with the decoy for the tail).

Of the 44 females, seven were excluded from the analysis because they did not visit any of the males during the experiment. The dual choice test revealed no overall preference for one trait over the other, although the majority of the females preferred the T‐males (“tail females” hereafter, *N* = 24), while others preferred the B‐males (“beard females” hereafter, *N* = 13; see Fig. 2). Replicated trials with a third decoy male revealed consistency in female preference as indicated by the significant effect of female identity (Table 1). As expected, the absolute preference for T‐males and B‐males was affected by the presence of a decoy. In contrast, the relative preference for T‐males over B‐males did not vary according to the type of decoy (*F*_2,110_ = 0.030, *P* = 0.970; Table 2). The absolute preference for the two decoy types confirmed that the majority of the females preferred the tail, as the absolute preference for the tail decoy was clearly stronger than that for beard decoy (Fig. 3).

**Table 1:** Relative preference for the tail male according to the set of male options (experimental trial, three levels: binary, tail decoy trinary, and beard decoy trinary) in a) all females, and b) according to the preference in the binary test

	Type III SS	df	MS	*F*	*P*
(a) All females (*N* = 37)					
Experiment trial	0.002	2,72	0.001	0.012	0.988
Female identity	6.583	36,72	0.183	2.500	<0.001
(b) Preference in the binary test					
B‐male (*N* = 13)					
Experiment trial	0.781	2,24	0.390	4.888	0.017
Female identity	1.479	12,24	0.123	1.543	0.177
T‐male (*N* = 24)					
Experiment trial	0.411	2,46	0.205	4.375	0.018
Female identity	2.288	23,46	0.099	2.120	0.015

**Table 2:** GLM post hoc test (Tukey B) for the effect of the interaction between the initial preference in the binary experiment and the female preference in the trials with the two decoys. The difference refers to the mean difference in the arcsin transformed percentage of time females spent close to the stimulus groups

Preference in the binary exp.	Experiments			Difference ± SE	*P*
B‐male	Binary	vs.	DB‐male	−0.321 ± 0.11	0.024
			DT‐male	−0.274 ± 0.11	0.062
	DB‐male	vs.	DT‐male	0.047 ± 0.11	0.965
T‐male	Binary	vs.	DB‐male	0.165 ± 0.06	0.033
			DT‐male	0.155 ± 0.06	0.051
	DB‐male	vs.	DT‐male	−0.011 ± 0.06	0.946

**Figure 4:**
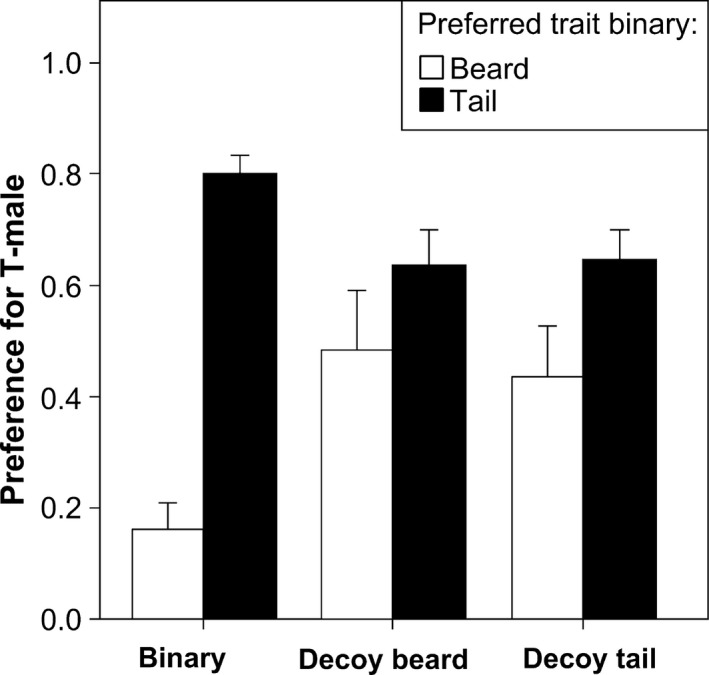
Relative preference for the T‐male in the three different experimental trials (binary choice; trinary with the decoy for the beard; trinary with the decoy for the tail). In white bars: females that preferred B‐male in the binary choice test. In black bars: females that preferred T‐male in the binary choice test.

**Figure 5:**
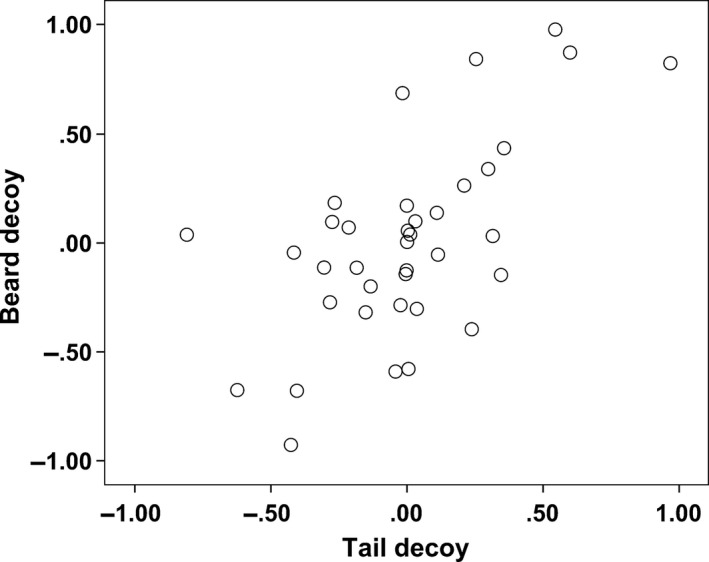
Relationship between the change in the relative preference for the tail in the presence of the beard decoy and the change in the relative preference for the tail in the presence of the tail decoy (expressed as the relative preference for the tail male in the presence of a decoy minus the relative preference for tail male in the binary test). The two variates were significantly correlated (Pearson correlation, *r* = 0.630, *P* < 0.001, *n* = 37).

**Figure 6:**
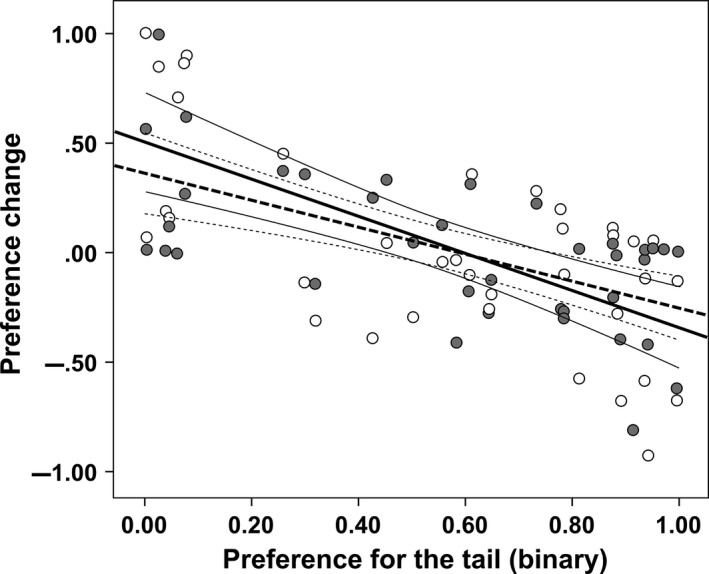
Change in the relative preference for the tail in the presence of a decoy in relationship with the relative preference for the tail in the binary tests and the type of decoy (white dots = beard decoy; gray dots = tail decoy). The relative preference was calculated as the time near the tail male over the total time near the tail and the beard male. The change in preference was expressed as the relative preference for the tail male in the presence of a decoy minus the relative preference for the tail male in the binary test. Positive values of the *y*‐axis indicate that the relative preference for the tail male was stronger than in the binary test. The lines represent the least square regression (continuous line = beard decoy: regression coefficient = −0.835 ± 0.166 SE,* t*_35_ = 5.043, *P* < 0.001; dotted line = tail decoy: regression coefficient = −0.608 ± 0.132 SE,* t*_35_ = 4.615, *P* < 0.001) and their 95% CI.

When the relative preference for T‐males over B‐males was separately analyzed for “tail” and “beard” females, we found that the presence of the decoy male significantly decreased their binary preference (Fig. 4, Table 2). This change was irrespective of whether the female preferred tail or beard and of the type of decoy male (Fig. 5). This effect was not due to our arbitrary subdivision of the test females in two groups (“tail” and “beard” females). Indeed, a further analysis revealed that the change in the relative preference for T‐males over B‐males (i.e., the relative preference for T‐males when the decoy was present minus the relative preference for T‐males in the binary test) was negatively correlated with the binary preference for T‐males, irrespective of the decoy type (Fig. 6).

## Discussion

This experiment provides two key results. First, it shows that when females are presented with options that vary antagonistically with respect to two ornaments, some individuals prefer one trait while others the other trait. In other words, if females can choose between two options that are antithetical for two sexually selected traits, a mate preference polymorphism emerges. Second, our results show that female preference for the main male options were not independent from the presence of alternatives. Indeed, when the data from all tested females were analyzed, the presence of a third stimulus did not affect the relative preference between the two main stimuli. The magnitude of this preference change was positively correlated with the strength of the preference in the binary test, that is, the more the one option was preferred in the binary test, the larger was the change in the relative preference when a third option was available. The two types of decoy had the same effect on the relative preference between the two main stimuli. Indeed, the correlation between the initial strength of the preference for one of the two main options in the binary tests and the reduction in the relative preference for that option in the trinary tests did not differ according to the decoy type. This result was unexpected, as the addition of a third, asymmetrical dominated decoy male (which is inferior to the main option males, but asymmetrically so) is predicted to be accompanied by an increase in the preference for the option by which the decoy is dominated (Bateson and Healy 2005).

Given that the repeatability of each female's choice for male trait is very high in our population (see Repeatability of mate preference in bearded reedlings) and that we controlled for order effect (the order of presentation of a different set of males varied randomly), our results suggest that mate preference in bearded reedlings is highly context dependent (although whether the decoy is asymmetrically dominated by one or the other main option apparently did not affect the change in preference). We can exclude the possibility that the pattern observed was mainly due to preference for relatively novel phenotypes, because females that preferred the T‐male in the binary trials reduced their preference for the tail even when a third male with a long beard was present (the same was observed in the beard females' group). Contrary to our results, Royle et al. (2008), in a study on green swordtails (*Xyphophorus helleri*) found that female preference for body size and sword length in binary tests changed when an asymmetrical dominated decoy was added. This shift in preference away from the type of male preferred in the binary choice depended by the type of decoy and it was best explained by a preference for relatively novel phenotypes.

A more likely explanation of the results of our experiment is that a female's choice may be constrained by her cognitive abilities when she is simultaneously presented with several options varying for two uncorrelated traits. This effect is apparently more evident when a female shows a stronger binary preference. Females that in a binary context showed a less pronounced preference for either tail or beard, in contrast, were not particularly affected by the presence of a third option. This may be because these females mate randomly (in this case, there is no reason to expect a significant change of the binary preference in any direction). Alternatively, they may base their choice on another male traits that were not considered. Considering that tail and beard were experimentally manipulated, any other (unknown) quality trait is expected to be uncorrelated with postmanipulation tail and beard length in both the main options and the decoys. Hence, there is no reason to expect a directional change of the binary preference in response to the presence of a decoy. The experimental manipulation of the male traits used by females in their mate choice is the usual paradigm in mate choice experiments (Andersson 1994). However, in this context, it may be interesting to use male stimuli that vary naturally for the traits of interests (whose expression most likely covary with other traits actually influencing a female mating decision) to test whether the pattern found here is confirmed. Given that our results shown a clear shift in mate preference with a presence of a third stimulus (as previous studies did, e.g., Royle et al. 2008), future studies should address the generality of these findings in other species, by comparing female preference with two and more than two stimuli, either experimentally manipulating the expression of the traits or exploiting their natural variation among males. Whatever the explanation for our results, it is also important to note that in natural conditions, females often have the option to choose among more than two males. Results from dichotomous mate choice tests may therefore offer an oversimplified perception of preference functions (Edward 2015). The observed shifts in mate preference when more than two males are available could represent an important mechanism of maintenance of the genetic variation for male traits in the population, as it has been postulated by Bateson and Healy (2005). For example, the change of preference when more options are available may enhance mating options for lower quality males, given that trait evaluation becomes more complex and may lead to suboptimal mate choice decisions. This would consequently mean that in species where mate choice is based on multiple traits, the most attractive males should avoid social environments with multiple potential mating competitors independent of their attractiveness. However, this higher cost for the most attractive male may be compensated by benefits of aggregating, like an increased number of visiting potential mating partners or increased opportunities for extra‐pair copulations.

As observed elsewhere (Wagner 1998; Royle et al. 2008), most of the studies use binary choice test (although there are exceptions, e.g., Brooks 2000; Zanollo et al. 2014). Although logistically more difficult, more natural experimental settings in mate choice experiments are necessary and we therefore encourage future studies to investigate the mate preference using not only a binary choice test, but offering the test subject several alternatives.

In conclusion, our results indicate that in our bearded reedlings population exists a significantly repeatable female mate preference polymorphisms for either tail of beard feather length. This preference, however, is context dependent and is severely attenuated in the presence of a third stimulus. The reasons why this “preference dilution effect” occurs, however, clearly require further investigation.

## Conflict of Interest

None declared.
